# Neutrophil-to-lymphocyte ratio and mortality in the United States general population

**DOI:** 10.1038/s41598-020-79431-7

**Published:** 2021-01-11

**Authors:** Minkyo Song, Barry I. Graubard, Charles S. Rabkin, Eric A. Engels

**Affiliations:** Infections and Immunoepidemiology Branch, Division of Cancer Epidemiology and Genetics, National Cancer Institute, National Institutes of Health, 9609 Medical Center Drive, Bethesda, MD 6E204 USA

**Keywords:** Cancer epidemiology, Cardiovascular biology, Circulation, Immunology, Biomarkers, Cardiology, Diseases, Endocrinology, Medical research, Molecular medicine, Risk factors

## Abstract

The neutrophil-to-lymphocyte ratio (NLR) in peripheral blood reflects the balance between systemic inflammation and immunity and is emerging as a prognostic biomarker in many diseases, but its predictive role for mortality in the general population has not been investigated. We analyzed 1999–2014 National Health and Nutrition Examination Survey mortality-linked data, followed up until 2015. In participants aged > 30 with measurements of differential white blood cell counts, NLR was calculated and categorized into quartiles. Associations of increased NLR with overall or cause-specific mortality were assessed with Cox proportional hazard regression models, adjusted for potential confounders. Increased NLR was associated with overall mortality (hazard ratio [HR] 1.14, 95% confidence interval [CI] 1.10–1.17, per quartile NLR) and mortality due to heart disease (1.17, 1.06–1.29), chronic lower respiratory disease (1.24, 1.04–1.47), influenza/pneumonia (1.26, 1.03–1.54) and kidney disease (1.26, 1.03–1.54). NLR was associated with cancer mortality only in the first follow-up year (HR 1.48, 95% CI 1.11–1.98). The association with chronic lower respiratory disease mortality was stronger in individuals with prevalent lung diseases (HR 1.46, 95% CI 1.14–1.88, P_interaction_ = 0.01), while NLR showed positive associations with mortality from heart disease (1.21, 1.07–1.38) and cerebrovascular disease (1.30, 1.04–1.63) only among individuals without these conditions at baseline. NLR is associated with mortality overall and due to certain causes in the general population. Associations over short follow-up intervals and among individuals with conditions at baseline suggest effects of disordered inflammation and immunity on progression of those conditions, while other associations may reflect contributions to disease etiology.

## Introduction

Inflammation and immunity play a critical role in many chronic diseases^1–3^. The neutrophil-to-lymphocyte ratio (NLR), calculated as a simple ratio between the neutrophil and lymphocyte counts measured in peripheral blood, is a biomarker which reflects the balance between two aspects of the immune system: acute and chronic inflammation (as indicated by the neutrophil count) and adaptive immunity (lymphocyte count). NLR has been extensively evaluated^4^ and shown to be associated with outcome and predict disease course among patients with a variety of medical conditions including ischemic stroke^5,6^, cerebral hemorrhage^7,8^, major cardiac events^9,10^ and sepsis and infectious diseases^11^. Furthermore, in cancer patients higher NLR has been associated with poor prognosis^12^. These adverse associations may reflect the contributions of severe inflammation and poor immune function to the progression of these diseases.

By extension, NLR may be predictive of mortality in the general population, and thus the overall impact of inflammation and immunity on health^13^. Several studies have reported that higher NLR was significantly associated with overall mortality and mortality due to cardiovascular disease, but not with mortality due to cancer^14–17^. In addition, it is important to evaluate associations of NLR with cause-specific mortality over different intervals of follow-up time, because associations over short intervals after baseline measurement of NLR predominantly reflect the effects of disordered inflammation or immunity for people who already have these medical conditions. In contrast, associations over intervals of several years reflect effects of these processes on both the incidence of disease and survival after diagnosis. While some studies have assessed associations over different follow-up intervals for mortality due to cardiovascular disease, cancer or stroke^15,17^, no study has systematically assessed such associations for multiple diseases.

An improved understanding of the association of NLR with general population mortality will help clarify the role of inflammation and immunity in the development and outcomes of diverse medical conditions. We investigated NLR associations with overall mortality and, for a range of medical conditions, cause-specific mortality in a prospective analysis of a representative sample of the US general population. To distinguish effects of inflammation and immune dysfunction on incidence and progression of disease, we also assessed associations of NLR with cause-specific mortality across different intervals of follow-up time and among individuals with and without specified medical conditions at baseline.

## Methods

The National Health and Nutrition Examination Survey (NHANES) is a population-based health survey of adults and children in the US, conducted by the National Center for Health Statistics (NCHS)^18^. NHANES uses a multistage probability sampling design to produce a nationally representative sample of the non-institutionalized US population. The survey consists of questionnaires administered in the home followed by physical examinations in mobile examination centers (MECs). In this study, we assessed participants from eight 2-year NHANES cycles conducted during 1999–2014. We included adults ≥ 30 years old with peripheral blood white blood count (WBC) and differential measurements.

Demographic information, medical history, and cigarette smoking status were ascertained from questionnaire data. Body mass index (BMI) was calculated from the physical examination weight and height (kg/m^2^). We inferred the baseline presence of diseases corresponding to the causes of death that we evaluated and risk factors for these conditions through the questionnaire responses and from laboratory measures of diabetes and kidney disease (Supplementary Table S1).

Blood specimens were collected as part of physical examinations, and WBCs were measured in the MECs. The WBC parameters were determined using the Beckman Coulter method of counting and sizing, and the automated differential was based on cell volume, conductivity, and light scatter technology^19^. We then calculated the NLR as the ratio of the derived neutrophil and lymphocyte counts.

NCHS previously linked NHANES participants with death certificate records from the National Death Index. We used the 1999–2014 NHANES public-use linked mortality files which provided mortality follow-up through December 31, 2015. Mortality outcomes in the public-use files include the following nine leading underlying causes of death (Tables 2, 3, 4): heart disease, cancer, chronic lower respiratory disease, unintentional injuries, cerebrovascular diseases, Alzheimer’s disease, diabetes, influenza and pneumonia, and kidney disease.

Statistical analyses were performed using pre-specified weighting that reflects the differential rates of participant selection as determined by the sample design and the inclusion of multiple NHANES surveys^19^. We used multiple variable Cox regression to assess NLR associations with overall and cause-specific mortality. Hazard ratios (HRs) were adjusted for age at baseline (i.e., MEC visit; continuous variable); sex; race/ethnicity (non-Hispanic white, non-Hispanic black, Mexican American, other Hispanic, other race/ethnicity); smoking (never, former, current smoker), BMI (< 18.5, 18.5–24.9, 25–29.9, 30+ kg/m^2^); baseline presence of diabetes mellitus, hypertension, and arthritis; and total WBC count. Follow-up time from the baseline visit was used as the timescale. NLR was categorized into quartiles based on the weighted distribution of results (NLR < 1.54, 1.54–2.00, 2.01–2.67, 2.68+). p values for trend across NLR quartiles were estimated using NLR quartile coded as an ordinal variable (values of 1, 2, 3 and 4) with 1-degree of freedom. We examined non-linear associations of NLR with mortality with restricted cubic spline functions using three knots placed at the 5th, 50th and 95th percentiles (NLRs of 1.00, 2.00 and 4.22)^20^.

We conducted two secondary analyses to further characterize NLR associations with mortality. First, we assessed associations between NLR and mortality outcomes within different time periods after baseline, with intervals categorized as the first year (0–11 months) and the rest of the follow-up period equally divided into tertiles based on the overall number of deaths (12–49, 50–93, and 94+ months) and tested for interactions between follow-up period and NLR (this interaction test corresponds to a test for proportional hazards). Presentation of results for cells with < 11 deaths were suppressed because of concerns about accuracy of the p values and confidence intervals (CIs). Consequently, for analyses with < 11 deaths in the 0–11-month period, p interactions were estimated for only the categories during 12+ months of follow-up.

Second, we explored whether the documented presence of a specific disease at baseline could affect the associations between NLR and mortality from that disease. We describe the distribution of times from baseline to death due to specified causes in individuals with or without those conditions at baseline. We then assessed HRs relating NLR to cause-specific mortality for diseased and non-diseased individuals, and we tested for heterogeneity by testing for an interaction between disease status and NLR.

All tests of statistical significance were based on two-sided p values of 0.05 and 95% CIs without adjustment for multiple comparisons. Analyses used SAS version 9.4 (SAS Incorporated, Cary, NC, USA) and its survey procedures to account for the complex sample design of NHANES.

### Consent to participate

The Institutional Review Board approval and documented consent was obtained from all participants for the use of NHANES data^21^. We confirm that all methods were carried out in accordance with relevant guidelines and regulations.

## Results

A total of 32,454 individuals were included in the study (Table 1). The mean age was 52.0 years at baseline, 48% were males, and 72% were non-Hispanic whites. Forty-eight percent were past or current smokers and 70% were overweight/obese. The prevalence of medical conditions of interest ranged from 10% (diabetes mellitus) to 35% (hypertension). The mean WBC count was 7.2 thousand cells/µl with mean neutrophil count of 4.3 thousand cells/µl and mean lymphocyte count of 2.1 thousand cells/µl. The mean NLR was 2.3. As shown in Table 1, compared with individuals with the lowest quartile of NLR, those with higher quartiles of NLR were older; more likely to be male, non-Hispanic whites, and current or former smokers; and more frequently to report the ascertained baseline medical conditions. Total WBC counts were also higher in participants with higher NLR quartiles.

**Table 1:** Baseline characteristics of the study population, overall and according to neutrophil-to-lymphocyte ratio quartiles (NHANES, 1999–2014).

Characteristic^a^	All	Q1	Q2	Q3	Q4
Age in years, mean (SD)	52.0 (14.4)	50.4 (13.0)	50.8 (14.0)	51.7 (14.7)	54.9 (15.5)
Male, N (%)	15,788 (47.6)	4083 (45.0)	3679 (47.2)	3766 (47.9)	4260 (50.2)
**Race/ethnicity**					
Non-Hispanic white	15,883 (72.2)	3092 (60.6)	3742 (72.5)	4112 (75.8)	4937 (79.8)
Non-Hispanic black	6414 (10.3)	2983 (19.4)	1329 (8.6)	1145 (7.4)	957 (5.6)
Mexican American	5570 (6.9)	1430 (7.5)	1491 (7.5)	1427 (7.1)	1222 (5.5)
Other Hispanic	2373 (4.9)	682 (5.6)	632 (5.4)	548 (4.7)	511 (4.0)
Other/multiple	2214 (5.8)	681 (7.0)	585 (6.0)	465 (5.0)	483 (5.0)
**Smoking status, N (%)**					
Non-smoker	16,676 (51.4)	4811 (53.7)	4154 (52.7)	3939 (52.4)	3772 (46.7)
Former smoker	9131 (27.7)	2261 (25.9)	2127 (27.7)	2124 (26.2)	2619 (30.8)
Current smoker	6615 (20.9)	1785 (20.2)	1492 (19.6)	1626 (21.3)	1712 (22.5)
Missing	32 (0.1)	11 (0.1)	6 (0.0)	8 (0.1)	7 (0.0)
**Body mass index in kg/m**^**2**^**, N (%)**					
< 18.5 (underweight)	417 (1.3)	112 (1.3)	85 (1.2)	89 (1.2)	131 (1.6)
18.5–24.9 (normal)	8365 (27.4)	2240 (27.9)	1991 (28.0)	1930 (25.9)	2204 (27.7)
25–29.9 (overweight)	11,313 (34.7)	3195 (35.6)	2762 (35.5)	2641 (33.9)	2715 (33.6)
≥ 30 (obese)	11,695 (35.0)	3177 (34.0)	2829 (34.0)	2901 (37.4)	2788 (34.7)
Missing	664 (1.6)	144 (1.2)	112 (1.3)	136 (1.6)	272 (2.4)
**Baseline medical condition, N (%)**					
Diabetes mellitus	4374 (9.8)	1089 (8.8)	952 (8.3)	1050 (10.2)	1283 (12.1)
Hypertension	12,867 (35.2)	3338 (32.8)	2854 (32.1)	3047 (35.6)	3628 (40.1)
Cancer	3419 (10.7)	694 (8.5)	711 (9.5)	797 (10.6)	1217 (14.1)
Arthritis	10,162 (28.9)	2506 (26.2)	2282 (27.4)	2425 (28.8)	2949 (33.3)
Cardiovascular/cerebrovascular disease	4299 (10.4)	864 (8.2)	877 (9.0)	1008 (9.9)	1550 (14.7)
Lung disease	5444 (17.4)	1349 (15.5)	1251 (17.3)	1292 (17.6)	1552 (19.3)
Kidney disease	5391 (13.6)	1318 (11.9)	1070 (11.1)	1203 (13.2)	1800 (18.1)
**White blood cell counts in thousand cells/µl, mean (SD)**					
Total	7.2 (2.3)	6.5 (2.4)	6.8 (1.8)	7.4 (2.0)	8.1 (2.5)
Neutrophils	4.3 (1.7)	3.1 (0.9)	3.9 (1.1)	4.6 (1.3)	5.7 (2.0)
Lymphocyte	2.1 (1.1)	2.6 (1.7)	2.2 (0.6)	2.0 (0.6)	1.6 (0.5)
Neutrophil-to-lymphocyte ratio, mean (SD)	2.3 (1.2)	1.2 (0.2)	1.8 (0.1)	2.3 (0.2)	3.7 (1.4)

*SD* standard deviation.

^a^N corresponds to the unweighted sample size. Percentages, means, and standard deviations are weighted to reflect sample design, participation rates, and the inclusion of multiple NHANES surveys (see Methods).

**Figure 1:**
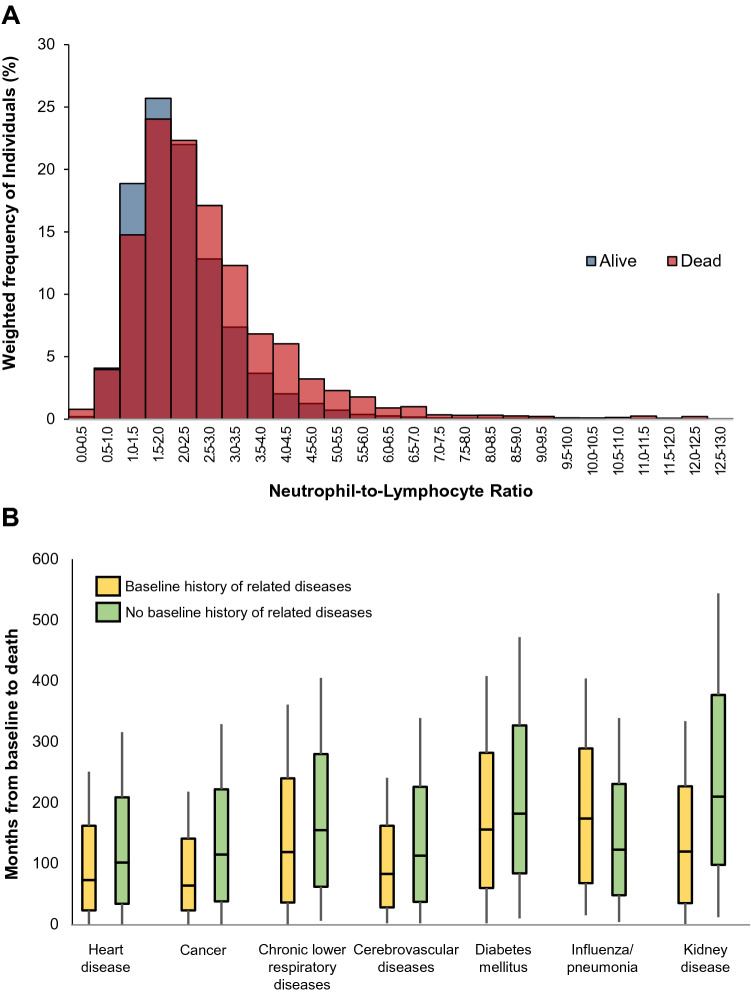
(**A**) Distribution of neutrophil-to-lymphocyte ratios among individuals according to vital status on follow up date. Results are weighted using NHANES survey sample weights. The x-axis is truncated at an NLR of 13, and not included in the graph are N = 11 surviving participants (weighted percentage 0.03%) and N = 11 deceased (0.20%) with greater values. (**B**) Follow-up time (months) in individuals who died from specified diseases, with or without those diseases at baseline.

A total of 4975 deaths occurred during 3,008,113 person-years of follow-up (median 86 months, interquartile range 48–135). Figure 1A shows the weighted distribution of baseline NLR values according to vital status at the last follow-up. Individuals who died had higher NLRs compared to those alive, with median NLRs of 2.33 vs*.* 1.99, respectively, although the overlap was substantial. With regards to specified causes of mortality, we observed that deaths from various medical conditions occurred earlier during follow-up among individuals who had baseline evidence for those conditions than in those without the conditions (Fig. 1B). The number of deaths by cause for each NLR quartile category is shown in Supplementary Table S2.

Table 2 describes associations of NLR with mortality. Increasing NLR was significantly associated with higher overall mortality (HR 1.14, 95% CI 1.10–1.17, per quartile of NLR). Higher NLR was also significantly associated with mortality due to specific causes including heart disease (HR 1.17, 95% CI 1.06–1.29, per quartile of NLR), chronic lower respiratory diseases (1.24, 1.04–1.47), influenza/pneumonia (1.26, 1.03–1.54), and kidney diseases (1.62, 1.21–2.17). For Alzheimer’s disease, quartiles 2, 3, and 4 of NLR were associated with elevated mortality, compared with quartile 1, but the linear trend was not significant (p = 0.06). Also, there were no notable associations of NLR with mortality due to cancer, cerebrovascular disease, accidents, or diabetes mellitus. We did not identify any nonlinear associations of NLR with overall or cause-specific mortality in models that included cubic regression splines (Supplementary Figure S1).

**Table 2:** Associations of neutrophil-to-lymphocyte ratio with overall and cause-specific mortality.

	Deaths, N	Per Q increase		Q2 *vs.* Q1	Q3 *vs.* Q1	Q4 *vs.* Q1
		HR (95% CI)	P_trend_	HR (95% CI)	HR (95% CI)	HR (95% CI)
Overall mortality	4975	**1.14 (1.10–1.17)**	** < 0.0001**	1.04 (0.94–1.16)	1.03 (0.91–1.16)	**1.45 (1.31–1.61)**
**Cause-specific mortality (ICD10 codes)**						
Heart disease (I00-I09, I11, I13, I20-I51)	900	**1.17 (1.06–1.29)**	**0.002**	1.09 (0.81–1.46)	1.10 (0.82–1.47)	**1.57 (1.15–2.14)**
Cancer (C00-C97)	1056	1.06 (0.98–1.14)	0.16	0.80 (0.63–1.01)	**0.76 (0.59–0.98)**	1.13 (0.92–1.39)
Chronic lower respiratory diseases (J40-J47)	197	**1.24 (1.04–1.47)**	**0.02**	1.56 (0.90–2.69)	1.59 (0.86–2.96)	**2.09 (1.17–3.74)**
Accidents (V01-X59, Y85-86)	109	0.78 (0.60–1.00)	0.05	1.17 (0.52–2.64)	0.73 (0.33–1.60)	0.47 (0.19–1.16)
Cerebrovascular diseases (I60-I69)	209	1.16 (0.96–1.40)	0.13	1.92 (1.08–3.39)	1.35 (0.68–2.70)	**1.96 (1.09–3.52)**
Alzheimer's disease (G30)	124	1.17 (0.99–1.39)	0.06	**2.23 (1.02–4.87)**	**2.12 (1.08–4.17)**	**2.17 (1.12–4.21)**
Diabetes mellitus (E10-E14)	132	1.04 (0.84–1.29)	0.71	0.93 (0.48–1.82)	1.11 (0.52–2.37)	1.08 (0.54–2.16)
Influenza/pneumonia (J09-J18)	82	**1.26 (1.03–1.54)**	**0.03**	1.71 (0.88–3.30)	1.27 (0.56–2.89)	**2.31 (1.33–4.02)**
Kidney disease (N00-N07, N17-N19, N25-N27)	89	**1.62 (1.21–2.17)**	**0.001**	1.76 (0.59–5.31)	2.48 (0.80–7.69)	**4.37 (1.44–13.28)**

Models are adjusted for age at baseline (continuous); sex; race/ethnicity (non-Hispanic white, non-Hispanic black, Mexican American, other Hispanic, other race/ethnicity); smoking (never, former, current smoker), body mass index (< 18.5, 18.5–24.9, 25–29.9, ≥ 30 kg/m^2^); baseline presence of diabetes mellitus, hypertension, and arthritis; and total white blood cell count, except for the model for diabetes mellitus-specific mortality, which did not include adjustment for diabetes mellitus.

Bolded values indicate statistical significance.

*CI* interval, *HR* hazard ratio, *Q* quartile.

We also assessed NLR associations within different time periods of follow-up (equivalent to an evaluation of the proportional hazards assumption; Table 3). There was a suggestion that the association of NLR with overall mortality was strongest in the first 12 months of follow-up (HR 1.39, 95% CI 1.23–1.57, per quartile of NLR), with lower HRs (95% CIs) of 1.17 (1.11–1.24), 1.18 (1.11–1.24), and 1.03 (0.98–1.08) for 12–49, 50–93, and 94+ months after baseline, but this trend was not statistically significant (P_interaction_ = 0.052). Similarly, although there was no association of NLR with cancer mortality for the overall follow-up, the HR was highest in the first 12 months (HR 1.48, 95% CI 1.11–1.98, per quartile of NLR) with a decline in strength over time (P_interaction_ = 0.041). The association of NLR with cerebrovascular disease mortality increased over time (P_interaction_ = 0.033), although no HR was significant in any interval. Although limited by case counts in the first 12 months after baseline, HRs for influenza/pneumonia appeared to decline over time across the remaining period (P_interaction_ = 0.047). There was no significant variation of HRs over time for heart disease, chronic lower respiratory disease or kidney disease.

**Table 3:** Association of neutrophil-to-lymphocyte ratio with overall and cause-specific mortality for different time periods of follow-up.

	0–11 months	12–49 months	50–93 months	94+ months	**P**_**interaction**_
	HR (95% CI)	HR (95% CI)	HR (95% CI)	HR (95% CI)	
Overall mortality	**1.39 (1.23–1.57)**	**1.17 (1.11–1.24)**	**1.18 (1.11–1.24)**	1.03 (0.98–1.08)	0.052
**Cause-specific mortality (ICD10 codes)**					
Heart disease (I00–I09, I11, I13, I20–I51)	1.15 (0.92–1.45)	**1.27 (1.08–1.50)**	**1.31 (1.11–1.54)**	0.97 (0.85–1.10)	0.785
Cancer (C00–C97)	**1.48 (1.11–1.98)**	1.12 (0.97–1.30)	1.07 (0.94–1.22)	0.90 (0.77–1.04)	**0.041**
Chronic lower respiratory diseases (J40–J47)	–	**1.64 (1.00–2.44)**	1.34 (0.98–1.82)	1.04 (0.82–1.32)	0.127*
Accidents (V01–X59, Y85–86)	–	0.74 (0.50–1.09)	0.64 (0.47–0.88)	0.95 (0.64–1.40)	0.201*
Cerebrovascular diseases (I60–I69)	0.81 (0.49–1.35)	1.06 (0.76–1.48)	1.22 (0.90–1.66)	1.24 (0.99–1.56)	**0.033**
Alzheimer's disease (G30)	–	1.36 (0.85–2.18)	1.28 (0.92–1.79)	1.06 (0.89–1.25)	0.414*
Diabetes mellitus (E10–E14)	–	0.87 (0.44–1.73)	0.84 (0.61–1.15)	1.14 (0.84–1.54)	0.349*
Influenza/pneumonia (J09–J18)	–	1.98 (0.93–4.22)	1.42 (0.97–2.08)	0.87 (0.66–1.15)	**0.047**
Kidney disease (N00–N07, N17–N19, N25–N27)	–	1.49 (0.79–2.82)	**2.56 (1.49–1.33)**	1.45 (0.99–1.20)	0.249*

Hazard ratios describe the change in mortality corresponding to a 1-quartile increase in neutrophil-to-lymphocyte ratio. Models are adjusted for age (continuous); sex; race/ethnicity (non-Hispanic white, non-Hispanic black, Mexican American, other Hispanic, other race); smoking (never, former, current smoker), body mass index (< 18.5, 18.5–24.9, 25–29.9, ≥ 30 kg/m^2^); baseline presence of diabetes mellitus, hypertension, and arthritis; and total white blood cell count, except for the model for diabetes mellitus-specific mortality, which did not include adjustment for diabetes mellitus.

Values not shown, indicated as “–“, for categories with number of deaths less than 11.

Bolded values indicate statistical significance.

*CI* confidence interval, *HR* hazard ratio, *Q* quartile.

*P_interaction_ corresponds to a global test for proportional hazards across the four periods, except for these outcomes indicated by *, where the interaction excludes the 0–11-month period.

Finally, in an additional set of analyses we assessed associations of NLR with overall mortality for individuals with or without specific diseases that were present at baseline (Table 4). HRs among individuals with baseline disease were significant for cancer (HR 1.18, 95% CI 1.05–1.32, per quartile of NLR), chronic lower respiratory disease (1.46, 1.14–1.88) and kidney disease (1.81, 1.32–2.47). In contrast, NLR showed positive associations with mortality from heart disease (HR 1.21, 95% CI 1.07–1.38, per quartile of NLR) and cerebrovascular disease (1.30, 1.04–1.63) only among individuals without those conditions at baseline. However, the interaction between NLR and preexisting disease was significant (P_interaction_ = 0.01) only for chronic lower respiratory disease.

**Table 4:** Associations of neutrophil-to-lymphocyte ratio with cause-specific mortality in individuals with or without specific diseases at baseline.

	Baseline history of related diseases	No baseline history of related diseases	P_interaction_
	HR (95% CI)	HR (95% CI)	
**Cause-specific mortality (ICD10**)			
Heart disease (I00–I09, I11, I13, I20–I51)	1.06 (0.95–1.25)	**1.21 (1.07–1.38)**	0.23
Cancer (C00–C97)	**1.18 (1.05–1.32)**	1.00 (0.91–1.10)	0.08
Chronic lower respiratory diseases (J40–J47)	**1.46 (1.14–1.88)**	1.04 (0.85–1.27)	**0.01**
Cerebrovascular diseases (I60–I69)	0.95 (0.70–1.29)	**1.30 (1.04–1.63)**	0.07
Diabetes mellitus (E10–E14)	1.04 (0.81–1.33)	1.26 (0.76–2.08)	0.96
Influenza/pneumonia (J09–J18)	1.21 (0.82–1.79)	1.27 (0.96–1.67)	0.91
Kidney disease (N00–N07, N17–N19, N25–N27)	**1.81 (1.32–2.47)**	1.31 (0.78–2.22)	0.44

Hazard ratios describe the change in mortality corresponding to a 1-quartile increase in neutrophil-to-lymphocyte ratio. Models are adjusted for age (continuous); sex; race/ethnicity (non-Hispanic white, non-Hispanic black, Mexican American, other Hispanic, other race); smoking (never, former, current smoker), body mass index (< 18.5, 18.5–24.9, 25–29.9, ≥ 30 kg/m^2^); baseline presence of diabetes mellitus, hypertension, and arthritis; and total white blood cell count, except for the model for diabetes mellitus-specific mortality, which did not include adjustment for diabetes mellitus.

Bolded values indicate statistical significance.

*CI* confidence interval, *HR* hazard ratio, *Q* quartile.

## Discussion

In this prospective study of a representative sample of the US general population, we observed associations of NLR with subsequent overall mortality and mortality related to specific conditions, including heart disease, chronic lower respiratory disease, influenza/pneumonia, and kidney disease. The NLR association with overall mortality appeared strongest in the immediate 1-year period after the baseline blood measurement, and we also observed a strong association with cancer mortality within this period. NLR was significantly associated with cancer or chronic lower respiratory disease mortality in individuals with a prior history of these diseases, and with heart disease and cerebrovascular disease mortality in individuals without prior histories of these diseases. These associations with NLR were independent of total WBC, smoking, BMI, and the presence of other chronic medical conditions.

An increase in NLR is determined by an increase of neutrophils and/or reduction in lymphocytes. Neutrophils play a major role in inflammation in pathologic conditions such as microbial infection, chronic tissue damage, and cancer^22,23^. An increase in circulating neutrophils is thus suggestive of an acute or chronic inflammatory response. Lymphocytes generate adaptive immune responses to eliminate specific pathogens, infected cells, and, in some instances, premalignant or malignant cells^24,25^. Decreases in circulating lymphocytes occur in association with recent infection, use of certain medications including chemotherapy, and in some immune-related medical conditions, including rheumatoid arthritis^26^. Neutrophils and lymphocytes may also be jointly regulated through complex mechanisms. Chronic inflammation can stimulate the release of immunoregulatory granulocytic myeloid-derived suppressor cells from the bone marrow, which can increase to 10% of peripheral WBCs and suppress lymphocyte counts and function^16,27^. High levels of circulating neutrophils are associated with depressed activity of other immune cells such as T-lymphocytes and natural killer cells^28,29^.

Although higher NLR was associated with increased overall mortality, the overlap of baseline levels for deceased and surviving participants was large. As expected, mortality due to specific conditions tended to occur earlier in individuals with baseline evidence for that disease than in those without such evidence, indicating that early deaths were in large part related to pre-existing conditions. Associations of NLR with short-term mortality for certain conditions may predominantly reflect the effects of heightened inflammation or reduced immunity on progression of those conditions in individuals who had already been diagnosed or who had preclinical disease. In contrast, significant NLR associations with disease mortality in later follow-up still reflects progression but may also be more related to an etiologic role of inflammation or impaired immune function on the development of the disease.

In secondary analyses, we observed associations of NLR with cancer mortality within the first 12 months of follow-up and among individuals who had a history of cancer at baseline. These findings are most consistent with a role of heightened inflammation or depressed adaptive immunity in shaping survival outcomes after a cancer diagnosis, since deaths from cancer in the first year were unlikely to be related to incident diagnoses within such a short interval. Neutrophils may directly suppress antitumor immune responses or secrete tumor growth promoting factors, while tumor-infiltrating lymphocyte play a role in controlling tumor growth and preventing metastases^12,30^. Although we could not look at mortality related to specific cancers using the public-use NHANES data, previous studies have demonstrated that an elevated NLR is associated with poor survival after diagnosis for a wide range of cancers^12,31^. Only a few studies have examined cancer mortality or incidence in the general population. One cohort study reported a nonsignificant trend of increased cancer mortality in association with elevated NLR^17^. Several studies have assessed NLR in relation to lung cancer incidence or mortality in the general population or among high-risk patients, and most^32–34^ but not all^35^, have found positive associations.

The mechanisms underlying heart and cerebrovascular diseases overlap to some degree, and the similarity of our results for these two conditions support inflammation or reduced immune function contributing to both diseases. Specifically, we found that NLR was associated with both diseases over long intervals of follow-up (i.e., 12–49 and 50–93 months for heart disease, and 94+ months for cerebrovascular disease) and in individuals without these conditions at baseline. Other studies have reported increased cardiovascular or cerebrovascular disease mortality or incidence in the general population among individuals with higher NLR^14–17^. One study noted that NLR levels were positively associated with elevated mortality from cardiovascular disease for up to 8 years after baseline measurement^17^. These results suggest that NLR could reflect inflammatory/immune processes that shape both the development of heart and cerebrovascular diseases and adverse outcomes from these disorders. Neutrophils secrete inflammatory mediators which contribute to degeneration of blood vessel walls, while lymphocytes may act to inhibit atherosclerosis^36^. NLR has also been associated with progression of atherosclerosis and higher mortality in patients with various cardiovascular diseases^37,38^.

NLR levels showed associations with increased mortality from chronic lower respiratory disease and kidney disease, with stronger associations observed in secondary analyses among individuals with baseline histories of these conditions. In addition, we observed associations of NLR with these mortality outcomes out to 49 months and 93 months after baseline for chronic lower respiratory disease and kidney disease, respectively. A recent meta-analysis showed a predictive ability of NLR for acute exacerbations in patients with chronic obstructive pulmonary disease^39^. Similarly, the prognostic value of NLR in kidney disease progression has been reported^40,41^. Although we did not observe an overall linear trend, high quartiles of NLR (quartiles 2, 3, and 4 vs. 1) had elevated HRs for mortality due to Alzheimer’s disease. Furthermore, NLR showed a positive relationship with mortality from influenza/pneumonia, which may be explained if individuals with elevated NLR levels are predisposed to respond with excessive inflammation or are unable to mount appropriate adaptive immune responses to pulmonary infections. Of interest, recent data indicate that NLR is a strong predictor of mortality among hospitalized patients with novel coronavirus 2019 disease^42^. Finally, we did not have clinical information with regards to the severity or the extent of the diseases under evaluation, so we could not assess NLR-mortality associations according to clinical status. For example, cancer survivors who had successful resection may have differed in their systemic inflammatory or immune responses from inoperable cancer patients at advance stage.

NHANES is a nationally representative sample of the non-institutionalized US population. The comprehensive assessment of various risk factors in NHANES allowed us to reliably adjust our analyses for potential confounding factors. Furthermore, standardized data and blood collection and laboratory testing strengthen confidence in our results. To our knowledge, this is the first study to systematically investigate associations of NLR with overall mortality and multiple cause-specific mortality outcomes. Nonetheless, our study has several limitations. Although NHANES includes a large sample, the small number of deaths for some causes limits the statistical power. Some mortality outcomes (e.g., cancer-specific mortality) are heterogenous because the underlying diseases are heterogeneous, so that the association of NLR with specific outcomes may not have been captured. We relied on a single measurement of blood counts, and repeated measurements of NLR over time may provide additional information. Finally, mortality reflects both incidence of a disease and survival following diagnosis and treatment. We had data identifying a limited number of people who had diseases of interest at baseline and on the timing of deaths, but because we lacked data on the incidence of disease after baseline, we could not completely distinguish between associations of NLR with incidence and progression.

In conclusion, our study demonstrates that NLR is predictive of mortality in the US general population. Further studies are warranted to understand the relationship of NLR with the incidence of specific diseases as well as outcomes following diagnosis. There is a large range of NLR values and overlap with regards to overall mortality, but NLR may be clinically useful for risk stratification depending on its combination with other information in particular clinical scenarios, and longitudinal measurements may add predictive utility. Studies examining more specific WBC subsets may further increase our understanding of disease etiology and progression.

## Supplementary Information

Supplementary Information

## Data Availability

All data used are publicly available at NHANES website.

## Code availability

Code can be provided upon request.
