# Supplementary material for: Neutrophil-to-lymphocyte ratio and mortality in the United States general population
Source: Sci Rep. 2021 Jan 11;11:464. doi: 10.1038/s41598-020-79431-7 (PMC7801737; doi:10.1038/s41598-020-79431-7)
Supplement: Supplementary file 1 — Supplementary Information [file 41598_2020_79431_MOESM1_ESM.docx]

***Original Article***

**Neutrophil-to-Lymphocyte Ratio and Mortality**

**in the United States General Population**

**Minkyo Song ^1^, Barry I. Graubard^1^, Charles S. Rabkin^1^, Eric A. Engels^1^**

^1^Division of Cancer Epidemiology and Genetics, National Cancer Institute, National Institutes of Health, Bethesda, MD, USA;

**Running title:** NLR and Mortality

**Correspondence to:**Minkyo Song, M.D., Ph.D.

Address: Infections and Immunoepidemiology Branch

Division of Cancer Epidemiology and Genetics

National Cancer Institute

9609 Medical Center Drive, 6E204

Bethesda, MD 20892-9776

Phone: 240-276-7985

Fax: 240-276-7806

Email: [minkyo.song@nih.gov](mailto:minkyo.song@nih.gov)

**Supplementary Table S1**. Prior/prevalent diseases that are underlying conditions leading to cause-specific mortality

|  | **Prior/Prevalent Disease** | **Assessment** | **Definition in Detail** |
| --- | --- | --- | --- |
| Underlying cause of death (ICD10) |  |  |  |
| Heart disease (I00-I09, I11, I13, I20-I51) | Cardiovascular/cerebrovascular disease | Questionnaire | congestive heart failure, coronary heart disease, angina, heart attack, stroke |
| Cancer (C00-C97) | Cancer | Questionnaire | cancer |
| Chronic lower respiratory diseases (J40-J47) | Lung disease | Questionnaire | asthma, emphysema, chronic bronchitis |
| Accidents (V01-X59, Y85-86) | N/A | N/A | N/A |
| Cerebrovascular diseases (I60-I69) | Cardiovascular/cerebrovascular disease | Questionnaire | congestive heart failure, coronary heart disease, angina, heart attack, stroke |
| Alzheimer's disease (G30) | N/A | N/A | N/A |
| Diabetes mellitus (E10-E14)* | Diabetes mellitus | Questionnaire | diabetes mellitus |
|  |  | Measurement | HbA1c≥6.5% |
| Influenza/pneumonia (J09-J18) | Lung disease | Questionnaire | asthma, emphysema, chronic bronchitis |
| Kidney disease (N00-N07, N17-N19, N25-N27) |  | Measurement | high serum creatinine (male ≥ 1.2mg/dL, female ≥1.0mg/dL) or urine albumin/creatinine ratio ≥300 |

N/A not applicable.

* Diabetes mellitus was determined to be present if either the questionnaire or measurement information indicated its presence.

**Supplementary Table S2**. Number of deaths by cause by neutrophil-to-lymphocyte ratio quartiles

|  | **All** |  | **Q1** | **Q2** | **Q3** | **Q4** |
| --- | --- | --- | --- | --- | --- | --- |
| Overall mortality | 4,975 |  | 957 | 970 | 1,108 | 1,940 |
|  |  |  |  |  |  |  |
| Cause-specific mortality (ICD10 codes) |  |  |  |  |  |  |
| Heart disease (I00-I09, I11, I13, I20-I51) | 900 |  | 152 | 171 | 207 | 370 |
| Cancer (C00-C97) | 1,056 |  | 270 | 199 | 207 | 380 |
| Chronic lower respiratory diseases (J40-J47) | 197 |  | 28 | 42 | 42 | 85 |
| Accidents (V01-X59, Y85-86) | 109 |  | 23 | 32 | 29 | 25 |
| Cerebrovascular diseases (I60-I69) | 209 |  | 37 | 46 | 43 | 83 |
| Alzheimer's disease (G30) | 124 |  | 16 | 24 | 34 | 50 |
| Diabetes mellitus (E10-E14) | 132 |  | 30 | 28 | 32 | 42 |
| Influenza/pneumonia (J09-J18) | 82 |  | 13 | 15 | 14 | 40 |
| Kidney disease (N00-N07, N17-N19, N25-N27) | 89 |  | 11 | 15 | 25 | 38 |


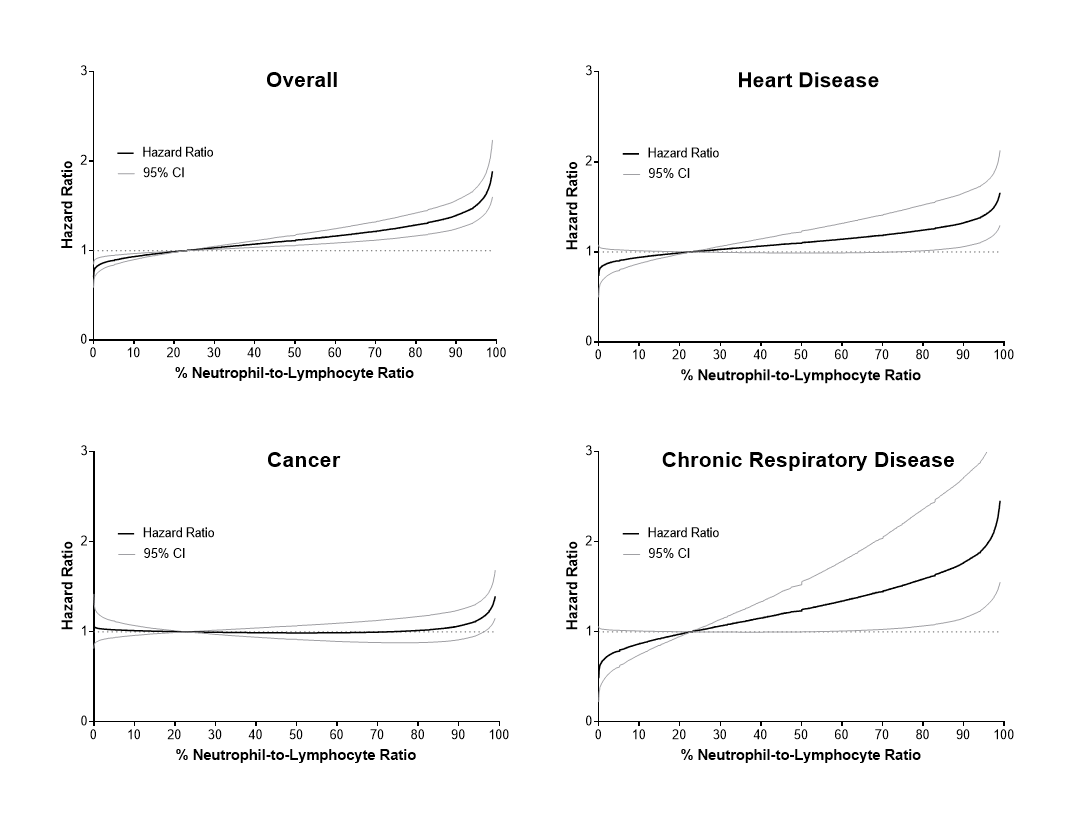


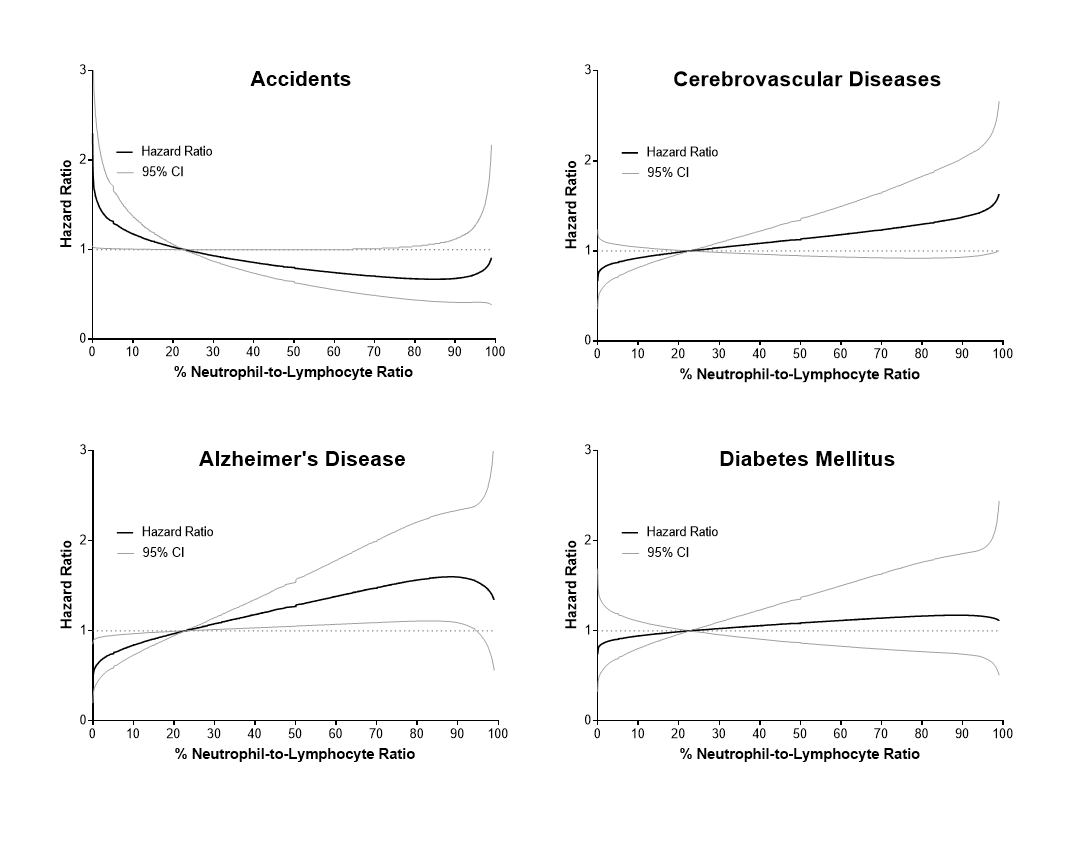

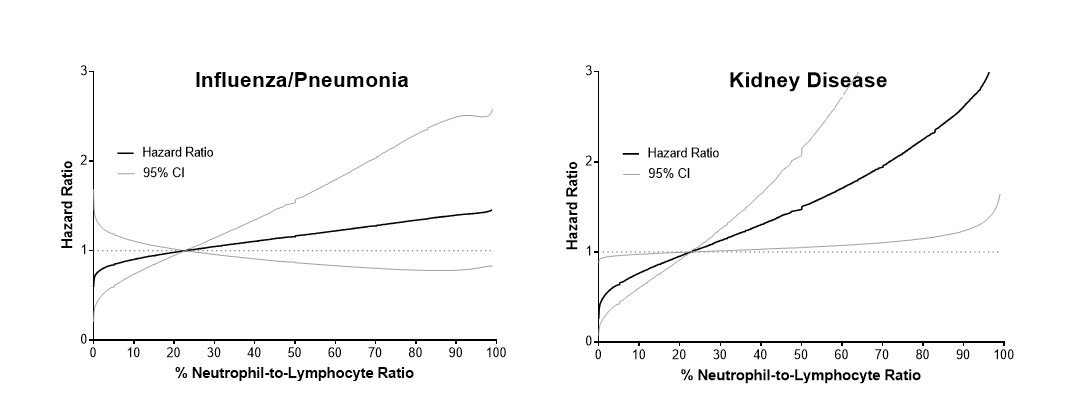
 **Supplementary Figure S1**. Hazard ratios for all-cause and cause-specific mortality by NLR percentiles in US adults

Models show the results for cubic splines for NLR and are adjusted for age (continuous); sex; race/ethnicity (non-Hispanic white, non-Hispanic black, Mexican American, other Hispanic, other race); smoking (never, former, current smoker), body mass index (<18.5, 18.5-24.9, 25-29.9, ≥30 kg/m^2^); baseline presence of diabetes mellitus, hypertension, and arthritis; and total white blood cell count, except for the model for diabetes mellitus-specific mortality, which did not include adjustment for diabetes mellitus.
